# When eyes outvote ears: Revisiting Tsay’s Sight-Over-Sound effect in music performance evaluation

**DOI:** 10.3389/fpsyg.2026.1767475

**Published:** 2026-04-28

**Authors:** Kilian Vogt, Gabriel Gutzmann, Reinhard Kopiez

**Affiliations:** Hanover Music Lab, Hanover University of Music, Drama and Media, Hanover, Germany

**Keywords:** audiovisual, audio-visual, evaluation, multi-modal, music competition, visual dominance

## Abstract

This study replicates Tsay’s Sight-Over-Sound (SOS) effect in music performance evaluation. The original study saw Tsay (2013) present participants with triads of performances originating from music competitions, including the eventual winner. In a three-alternative forced choice (3-AFC) task, participants had to identify the winning player. Participants performed better—to a level beyond that which chance (1/3) could explain—when the triads were presented as video-only, compared to audio-only or audiovisual presentations, suggesting a visual dominance or Sight-Over-Sound effect. The present study aimed to conceptually replicate this effect using excerpts from the semi-final rounds of four international violin competitions. The stimuli were controlled for musical content, camera perspective, and camera movement, and each set included the eventual winner. In an online experiment, 104 participants completed the 3-AFC task for two competitions in the audiovisual condition and for the remaining two competitions in both audio-only and video-only conditions, respectively. Responses were analyzed within a Signal Detection Theory framework. Participants performed above chance in the video-only condition (*M* = 50.5% correct, *d*′ = 0.572), but did not exceed chance in the audio-only (*M* = 35.1% correct) or audiovisual (*M* = 38.0% correct) conditions, respectively. Perceptual abilities measured by the Goldsmiths Musical Sophistication Index, as well as violin or viola expertise, predicted performance in the audio-only but not in the video-based conditions. The latter is consistent with both Tsay’s original study and previous replication attempts. Taken together, the present results further support the existence of a Sight-Over-Sound effect in music performance evaluation. The findings suggest that as well as a focus on preparing the auditory domain carefully, musicians should also take visual performance components into consideration.

## 1 Introduction

Music is often accompanied by processes of evaluation (Hasselhorn and Wolf, 2018). Such evaluations can be implicit, such as unconscious judgments about whether a piece, song or interpretation is liked or disliked, or explicit, such as who gets a position in an ensemble or wins a music competition. In formal settings such as examinations or music competitions, judges typically base their assessment on a set of musical parameters (McPherson and Schubert, 2022). However, this perspective is limited, given that “any music evaluation will be influenced by a number of additional factors that impact on … validity and reliability” (McPherson and Schubert, 2022, p. 104) as a general rule. These factors can be categorized as musical, extra-musical, non-musical, respectively, as well as measurement errors, though some aspects could fall into multiple categories depending on circumstances. Extra-musical factors include, among other things, visual aspects of the performance (McPherson and Schubert, 2022; Waddell and Williamon, 2022). Synthesizing 15 studies with a total sample size of 1,298 participants, Platz and Kopiez (2012) compared ratings of audiovisually presented music performances with audio-only presentations. Their meta-analysis yielded a medium effect size of Cohen’s *d* = 0.51, 95% CI [0.42, 0.59], for the visual component. Moreover, Platz and Kopiez (2022) conceptualize the live music concert as a “multisensory communicative setting that addresses all senses” (p. 84). Sensory inputs and their interactions are relevant when evaluating musical performances. Evidence for this can be found in the phenomenon of cross-modal effects or illusions (McPherson and Schubert, 2022; Platz and Kopiez, 2022), for example in the “McGurk effect” (originally observed for spoken syllables by McGurk and MacDonald, 1976) or in its musical variant (Schutz and Lipscomb, 2007). The communicative aspects of the live music concert setting include—but are not limited to—persuasion (e.g., Aronson et al., 2018, chap. 7) and impression management (Goffman, 2022).

A more in-depth review of the literature on the role of sight in listening reveals multiple interactions of senses, which also play a role in performance evaluation. For example, in their seminal study, Mershon et al. (1980) found that sound sources are often mislocalized to the nearest plausible visual target. Short auditory stimuli presented by a hidden loudspeaker were assigned to the visible dummy loudspeaker in about 90% of all cases. Results are interpreted as the “visual capture effect” which means that visual cues can override those of auditory distance. Another example from the speech perception domain is the ventriloquism effect, a psychological phenomenon where visual cues dominate auditory speech perception to make a sound appear as if it originates from a seen source. Vroomen and de Gelder (2004) and Woods and Recanzone (2004) examined this effect as an example of multisensory integration and argue that these cross-modal interactions are automatic and pre-attentive. They occur regardless of whether a person is consciously focusing on the stimuli. Overall, findings suggest that sensory modalities are not isolated but instead share an interactive relationship that shapes our experience of the world by merging auditory and visual information to create a unified and ecologically efficient representation of our environment. More recent research from the field of neuroscience (for an overview, see Lee and Wallace, 2019) revealed that brain regions once thought to be exclusively auditory are actually deeply affected by other sensory inputs to create a unified perceptual experience. This might provide a foundational understanding of how our senses work in concert to navigate the everyday world.

Even in the construction of concert halls, successful auditorium design must balance the auditory and visual domains to optimize the spectator experience, as both modalities contribute to the perceived intimacy and quality of a seat. As Jeon et al. (2008) identified, while a clear, unobstructed stage view and visual comfort are significant, the sound pressure level is the primary predictor of audience preference. The study highlights a multisensory interaction where high-quality acoustics can sometimes compensate for poorer sightlines, although auditory cues generally exert a much stronger influence on overall satisfaction than static visual images. Kawase (2013) also investigated the psychological motivations behind audience seat selection in concert halls, comparing the preferences of music- and non-music majors. Using a real-world seating map, the study revealed that concertgoers prioritize visual factors, such as the visibility of the performers, over auditory quality when choosing where to sit. While both groups shared similar seating patterns—preferring central spots for most ensembles and left-side views for piano soloists—music majors demonstrated higher sensitivity to sound quality and were more likely to identify specific trade-offs between aural and visual data. Ultimately, seat selection is a flexible strategy used by active listeners to enhance their personal experience of musical communication based on the genre and size of the performance.

Another line of research for a better understanding of the complex multisensory nature of the interaction of senses in music performance evaluation comes from the more recently developed discipline of Sensory Studies (Howes, 2022). This approach “treats the human sensorium as a dynamic whole that is best approached from historical, anthropological, geographic, and sociological perspectives … by directing attention to the sociality of sensation and the cultural mediation of sense experience and expression” (University of Toronto Press, 2026). Although primarily focused on the interaction between musicians, Maslen (2022) explores how professional musicians maintain multimodal perception to navigate the high-pressure, real-time demands of live performance. Because sound happens too quickly for players to rely solely on their ears for adjustments, they must hear ahead of the music by integrating auditory information with other physical cues, for example by watching the seen gestures of colleagues to synchronize entries and phrasing, internalizing a rhythmic pulse to feel the music’s progression, and utilizing proprioception to anticipate the quality of a note before it is even struck. As a result, the body’s internal sense of movement and external visual signals work together to ensure social and artistic cohesion. The successful coordination between ensemble musicians was also the subject of an ethnographic study by Pentimalli and Gobo (2023): Based on observations from a musical trio rehearsed with their hearing suppressed, the authors demonstrate that performers rely heavily on a phenomenon they call “visual hearing” which means the existence of hybrid senses that go beyond simple collaboration. This merged sensory experience allows musicians to “see” sound by monitoring pertinent visual cues, such as silent fingering, body sways, and breathing patterns, to maintain rhythm and manage transitions. Ultimately, the study shifts the focus from multisensoriality—where senses work alongside each other—to a hybridization of perception where the eyes effectively perform the work of the ears. Most of these assumptions on successful inter-performer communication also apply to the audience’s perception and performance evaluation.

Finally, in recent decades, numerous experimental studies have shed light on the complex and multimodal communicative setting of audiovisual music performance evaluation. For example, Vuoskoski et al. (2014) studied how expressive visual gestures and auditory cues combine to shape our perception of musical expressivity. The authors synchronized mismatched video and audio of piano performances to demonstrate that visual information often carries more weight than sound when audiences judge a performer’s intensity. While both modalities contribute to the experience, the study provides evidence of crossmodal interactions, where what we see can actually alter how we hear the music’s emotional character. In certain performance conditions, visual cues affected the ratings of auditory expressivity, and auditory cues had a small effect on the ratings of visual expressivity. Ultimately, the findings suggest that a musical performance is a multisensory phenomenon that is perceived as more than just the sum of its individual parts. Platz and Kopiez (2013) and Waddell and Williamon (2017) found that audience evaluation does not start with the first sound but is already influenced by the pre-performance or stage entrance phase. Expressive gestural communication might also positively influence the evaluation (Davidson, 1993; Schutz and Lipscomb, 2007; Behne and Wöllner, 2011), as well as the dress (Griffiths, 2008), or playing from score vs. by heart (Kopiez et al., 2017). Although Platz and Kopiez (2013) identified a set of six “golden rules” of pre-performance stage behavior that influence the audience’s motivation to continue performance elaboration, the main criterion from the performer’s perspective is the adequacy of behavior: Adequate stage behavior cannot be described by a catalog of dos and don’ts, but should always be considered in the context of performance venue, musical genre, and audience expectations (Griffiths, 2010). In other words: what is adequate for the jazz club might be inadequate for the chamber music concert (Huang and Krumhansl, 2011).

All this applies for music competitions, which can be considered a special concert category.

### 1.1 The Sight-Over-Sound effect

In a series of experiments, Tsay (2013, 2014) reported on the audiovisual interaction in live music competitions. Given the importance of Tsay’s studies for our study as reference points, the core results of her experiments will be summarized in the following sections.

In Experiment 1 of her first study, Tsay (2013) asked the participants to choose audio-only, video-only, or audiovisual recordings to judge the three finalists of each of 10 music competitions, then guess the winner. Based on a game-theoretical paradigm, correct guesses would be rewarded by an extra reimbursement, but this bonus would be reduced when choosing audiovisual compared to audio-only or video-only recordings. Tsay argued that the “tax on the video-plus-sound recordings, the option with most information, thus offers a strong test of beliefs about the judgment of performance” (2013, Supporting Information p. 2). Based on 58.5% of participants showing a preference for audio-only recordings and 27.4% for the audiovisual recordings, she concluded that most participants were convinced that sound outweighed visuals when it comes to evaluating music performances, but that both best approximate the circumstances under which the original jury selected the winner.

In Tsay’s (2013) Experiments 2 to 5, participants were presented with 6-s clips of the top three finalists from 10 international classical music competitions (predominantly involving piano). A triad of competition clips was presented as a three-alternative forced choice (3-AFC) task in which participants had to identify the winner, whereby the percentage of correct responses represented the performance criterion. A correct response was defined as selecting the performer whom the original jury had declared the winner.

In Experiment 2, in a within-subjects design, *N* = 106 participants with little to no experience of classical music were presented with 10 triads in both audio-only and video-only conditions. Their performance was hypothesized to be at chance—namely 1/3 or 33.3%—but a surprising result showed participants performing clearly above chance in the video-only condition, *M* = 52.5%, *t*(105) = 10.90, *p* < 0.001, but below chance in the audio-only condition, *M* = 25.5%, *t*(105) = −5.23, *p* < 0.001^1^.

In Experiment 3, Tsay (2013) added an audiovisual condition, that is, video recordings including sound, to test whether more information compared to the audio-only and video-only conditions would improve participant performance. This time, the conditions served as a between-subjects factor. The 10 triads were presented in either the audio-only, video-only, or audiovisual condition to *N* = 185 participants with little to no experience of classical music. Participants performed at chance in the audiovisual condition, *M* = 35.4%, *t*(67) = 0.94, *p* = n.s., below chance in the audio-only condition, *M* = 28.8%, *t*(66) = −2.09, *p* = 0.04, and above chance in the video-only condition, *M* = 46.4%, *t*(49) = 4.04, *p* < 0.001, respectively. Therefore, Experiment 3 replicated the findings from Experiment 2.

In Experiment 4, when Tsay (2013) applied the within-subjects design of Experiment 2 to *N* = 35 professional musicians, the results resembled those of Experiment 2: The participants performed below chance in the audio-only condition, *M* = 20.5%, *t*(34) = −6.11, *p* < 0.001, and above chance in the video-only condition, *M* = 46.6%, *t*(34) = 4.05, *p* < 0.001.

In Experiment 5, the between-subjects design of Experiment 3 was reused, assigning *N* = 103 professional musicians to either audio-only, video-only, or audiovisual condition. As with non-experts, participants performed at chance in the audiovisual condition, *M* = 29.5%, *t*(39) = −1.43, *p* = n.s., below chance in the audio-only condition, *M* = 25.7%, *t*(29) = −3.34, *p* = 0.002, and above chance in the video-only condition, *M* = 47.0%, *t*(32) = 3.4, *p* = 0.002.

In Experiment 6, *N* = 89 participants were presented with video-only clips that were reduced to movement outlines, isolating visual information to basic motion alone (akin to the vectorization of pictures in modern digital image processing). Even in this extremely reduced presentation mode, participants identified the winners above chance, *M* = 48.8%, *t*(88) = 6.49, *p* < 0.001.

In Experiment 7, the original task of identifying the winner was replaced by a set of six characteristic adjectives. For each adjective, participants had to choose one performer from the triad who demonstrated it most strongly (e.g., the most confident or creative performer). They were allowed to choose the same performer for all six adjectives, but this was not mandatory. In a between-subjects design, *N* = 262 participants were presented with the 10 triads in either audio-only or video-only condition. The participants’ choices for the adjectives were mapped against the original competition outcomes. For the video-only condition, the chosen performers matched the winner in 37.4% (“confident”) to 59.6% (“passionate”); for the audio-only condition, chosen performers matched the winner in 26.1% (“creative”) to 39.5% (“confident”). Besides the adjective “confident,” the chosen performers matched the winner significantly more often in the video-only condition than in the audio-only condition.

Besides testing against the chance level, Tsay (2013) also compared the conditions from Experiments 2–5 and 7 against each other. In all cases, prediction of the winner in the video-only condition was significantly better than in the conditions involving audio. For the comparison of the video-only and audio-only conditions, Tsay (2013) provided effect sizes ranging from Cohen’s *d* = 0.88 to *d* = 1.66.

In a follow-up study, Tsay (2014) reported on another set of between-subjects experiments replicating her previous findings. Again, triads of 6-s clips served as stimuli, but this time from the finals of group music competitions. In the first experiment, *N* = 118 non-expert participants were presented with eight triads in either audio-only, video-only, or audiovisual condition. The clips containing video showed the entire ensemble. In line with her 2013 study, participants performed at and below chance in the audiovisual and audio-only conditions, respectively, but above chance in the video-only condition, *M* = 46.4%, *t*(40) = 4.28, *p* < 0.001. For the comparison of the video-only and audio-only condition, Tsay (2014) provided an effect size of Cohen’s *d* = 1.16. In the second experiment, the same triads were used, but in the video-only condition only the ensemble leader was shown. Results based on *N* = 130 participants resembled a performance above chance in the video-only condition, *M* = 43.8%, *t*(50) = 4.90, *p* < 0.001, and at chance in the conditions involving audio.

In the third experiment, Tsay (2014) only used silent videos from the eight ensemble competitions. In the first of three conditions, the clips showed the entire ensemble; in the second condition, the clips showed the ensemble leader (e.g., the first violinist in a string quartet); in the third, the clips showed an ensemble member who was not the leader. Out of the *N* = 166 participants, those in the conditions showing the entire ensemble and the leader identified the winning group above chance, *M* = 47.8%, *t*(59) = 5.22, *p* < 0.001, and *M* = 43.2%, *t*(60) = 4.94, *p* < 0.001, respectively, whereas participants in the non-leader condition performed at chance, *M* = 33.4%.

In the fourth experiment, Tsay (2014) tested the performance of *N* = 283 participants in four conditions: audio-only, audiovisual (entire ensemble), video-only (entire ensemble), and video-only showing only the ensemble leader. Results resembled the previous three experiments on the same material. Only participants in the video-only condition showing the whole ensemble or the ensemble leader identified the winning group above chance, *M* = 55.3%, *t*(52) = 9.60, *p* < 0.001, and *M* = 41.4%, *t*(72) = 3.84, *p* < 0.001, respectively.

In the fifth experiment, Tsay (2014) used a different set of stimuli. She paired 6 s clips of performances by the top 10 ranked orchestras with matched clips by regional or university-related orchestras. The clips were presented to *N* = 172 participants either in an audio-only, video-only, or audiovisual condition. Using pairs rather than triads resulted in the two-alternative forced choice (2-AFC) task of selecting the top 10 group and a chance level of 1/2 or 50%. Although the quality differences within these pairs should have been more apparent than in the triads of finalist ensembles, participants in the audio-only condition performed at chance, *M* = 53.0%, *t*(55) = 1.30, *p* = n.s., whereas performance was above chance in the remaining two conditions, *M*_audiovisual_ = 60.6%, *t*(53) = 5.10, *p* < 0.001, and *M*_video-only_ = 64.3%, *t*(61) = 8.13, *p* < 0.001. The effect size for the comparison of the video-only and audio-only conditions was reported as Cohen’s *d* = 0.72.

In the sixth experiment, Tsay (2014) reused the design from the fourth experiment, but recruited *N* = 193 professional musicians as participants. Here, only participants in the video-only condition evaluating the entire ensemble performed above chance, *M* = 40.1%, *t*(37) = 2.30, *p* = 0.027, but not those in the video-only condition showing the ensemble leader, *M* = 35.8%.

Although the vast majority of the participants rated sound to be most important for the evaluation of music performances, they identified winning performances at or even below chance in the conditions involving sound. In contrast, given silent video clips, they were able to identify the winners near 50% of the time (Tsay, 2013, 2014). Taken together, Tsay (2013) hypothesized an effect of “visual dominance” (p. 14581) or—as named in the article title—a Sight-Over-Sound (SOS) effect.

### 1.2 Replication attempts

In recent years, attempts have been made to replicate the SOS effect, albeit with mixed results.

Mehr et al. (2018) reported three experiments on the SOS effect: In their first experiment, they used nine out of the 10 triads from Tsay (2013, Experiments 2–5, 7). Mehr et al. (2018) recruited *N* = 375 participants (*n* = 125 per condition) and assessed their musical expertise with the Musical Ear Test by Wallentin et al. (2010). Besides this continuous measure instead of a dichotomy of experts and non-experts, Experiment 1 by Mehr et al. (2018) represents a direct replication of Tsay’s (2013) Experiments 3 and 5. Analyses using one sample *t*-tests against chance level revealed that performance in the 3-AFC tasks was above chance in the video-only condition, *M* = 38.7%, *t*(124) = 3.55, *p* < 0.001, *d* = 0.32, but at chance in the audiovisual condition, *M* = 36.4%, *t*(124) = 1.81, *p* = 0.072, and below chance in the audio-only condition, *M* = 29.7%, *t*(124) = −2.79, *p* = 0.006. These results are consistent with the original study. They also persisted in a sub-sample of participants with very high scores on the Musical Ear Test. In an ordinal logistic regression analysis, the Musical Ear Test Scores did not predict the percentage of correct responses in any of the three conditions. The absence of an expertise effect is also in line with the original study.

In their second experiment, Mehr et al. (2018) used new stimulus material. Stimuli consisted of pairs of finalists resulting in a 2-AFC task. Additionally, six-second excerpts other than those from Experiment 1 were used. Again, the performance of the participants (*N* = 300, *n* = 100 per condition) was tested against chance level—which is 50% for a 2-AFC task—based on *t*-tests. While the results for the audio-only and audiovisual conditions matched those from Experiment 1, in contrast to Experiment 1 and Tsay’s (2013) results, performance in the video-only condition did not exceed chance level, *M* = 51.1%, *t*(99) = 0.64, *p* = 0.53. As in Experiment 1 there was no relationship between the Musical Ear Test score and the performance in identifying the winner.

In their third experiment, Mehr et al. (2018) omitted the Musical Ear Test but again used different material. Competition winners were paired with musicians eliminated in earlier rounds to possibly achieve a more salient difference within a pair. Based on a total sample of *N* = 150 (*n* = 50 per condition), the pattern of results was contrary to those from Experiment 1 and the original study: Performance was above chance for the conditions involving audio, *M*_audio-only_ = 68.4, *t*(49) = 6.70, *p* < 0.001, *d* = 0.95, and *M*_audiovisual_ = 63.6, *t*(49) = 5.00, *p* < 0.001, *d* = 0.71, but did not differ significantly from chance in the video-only condition, *M* = 45.2%, *t*(49) = 1.80, *p* = 0.078. In summary, in the experiments reported by Mehr et al. (2018), the direct replication was successful, whereas the two conceptual replication attempts failed to replicate the SOS effect.

Wilbiks and Yi (2022) attempted to directly replicate Tsay’s (2014) findings on ensemble competitions in the video-only condition. Following a power analysis, they recruited *N* = 96 musically non-expert participants. The original ensemble triads from Tsay (2014) were used as stimuli. With a mean of *M* = 35.0% correct responses and based on frequentist and Bayesian *t*-tests, Wilbiks and Yi (2022) conclude that their participants were unable to identify the winners above chance.

Chiba et al. (2023) attempted a conceptual replication of the SOS effect. They criticized that Mehr et al. (2018) had only used one-sample *t*-tests against chance level and did not compare the conditions with each other (i.e., pairwise *t*-tests). A reanalysis of the data from the second experiment reported by Mehr et al. (2018) revealed a significantly better performance in the video-only condition compared to the audio-only condition. In their own experiment, Chiba et al. (2023) applied a 2-AFC task. They used pairs from the five piano competitions from Tsay’s (2013) original study and five pairs from the second experiment reported by Mehr et al. (2018). This was done to introduce quality variance as an experimental factor with the pairs from the original study and the pairs by Mehr et al. (2018) constituting low- and high-variance conditions, respectively. Chiba et al. (2023) further expanded their experimental design by adding five pairs of low- and high-variance Tsugaru shamisen (a Japanese folk instrument) performances from different competitions. This resulted in a 2 (condition: audio-only vs. video-only) × 2 (instrument: piano vs. Tsugaru shamisen) × 2 (variance: low vs. high variance) within-subjects design. They hypothesized an interaction effect of condition and variance, expecting performance to be better for low-variance pairs in the video-only condition than in the audio-only condition, and vice versa for high-variance pairs.

The analysis of *N* = 155 participants resulted in the hypothesized interaction effect being significant for both instruments. Moreover, the prediction for the low-variance condition (i.e., video-only > audio-only) was confirmed for piano pairs, and the prediction for the high-variance condition (i.e., audio-only > video-only) was confirmed for the Tsugaru shamisen pairs. The predictions for the piano in the high-variance condition (i.e., audio-only > video-only) and for the Tsugaru shamisen in the low-variance condition (i.e., video-only > audio-only) could not be confirmed. Chiba et al. (2023) concluded that their results are in line with Tsay (2013) and Mehr et al. (2018) in that they confirm the SOS effect for the piano stimuli and that performance in the 2-AFC task is modulated by the variance within the pair.

Samma et al. (2025) attempted to conceptually replicate the SOS effect using material from Japanese brass band competitions. They used 30 performances from the final round of regional high school competitions and compiled 10 triads with one of the three bands having qualified for the national All Japan Brass Band Competition. Clips were limited to 6 s. Using a between-subjects design, the triads were presented to *N* = 301 participants, who were classified as brass band musicians, non-brass band musicians, and non-musicians, in either audio-only, video-only, or audiovisual condition. Based on a Kruskal–Wallis test, Samma et al. (2025) reported no significant difference between the three conditions, however, the participants in the video-only condition (*n* = 97) performed better than chance, *M* = 40.9%, based on a one-sample Wilcoxon signed-rank test, *V* = 3,568, *p* < 0.001, *r* = 0.40. They also conducted sub-sample analyses. For the brass band musician sub-sample (52–61 participants per condition), no differences between conditions or from chance level were found. In the non-brass band musician sub-sample, participants in the video-only condition performed significantly better than those in the other two conditions and than chance, *M* = 49.5%. Although the data in the non-musician sub-sample trended as expected by the SOS effect (*M*_video-only_ = 43.2 > *M*_audio-only_, *M*_audiovisual_), no significant differences from chance level or between the conditions emerged. Arguing that the findings suggest the presence of the SOS effect only in the non-brass band musician sub-sample, Samma et al. (2025) conclude that the replicability of the effect depends on the stimuli—namely, control for camera and musical content, variance between the performers within a triad or pair, whether ensembles or soloists are evaluated—and the participants’ own musical experience.

To summarize, the previous replication attempts show mixed results but also differ in their methods (e.g., statistical analyses comparing the conditions against chance level vs. comparing them against each other, stimulus material from soloists vs. ensembles and from different instruments). Some of them replicated the SOS effect, whereas others failed to. This applies to both verbatim and conceptual replications, raising questions about both the stability and generalizability of the effect.

### 1.3 Study aims

The main aim of our study was to conceptually replicate the SOS effect in the evaluation of soloists. Therefore, we expect the number of correct responses in the video-only condition to exceed chance level, whereas the number of correct responses in the audio-only and audiovisual conditions is not expected to differ from chance level. Furthermore, participants’ performance in the video-only condition is also expected to exceed that in the conditions involving audio.

This study addresses the issue concerning the stimuli for the soloist case as expressed by Mehr et al. (2018), which was addressed by Samma et al. (2025) for the ensemble case, for the soloist case: Rather than using stimulus material that differs in terms of the piece of music or the camera perspective and movement within a competition, our objective was to strictly control for these potential confounding factors in the selection of material.

We also employed a more sophisticated analysis strategy other than *t*-tests, which better reflects the comprehensive character of the responses obtained from a 3-AFC paradigm.

Moreover, previous studies differ regarding the role of musical experience in participants. Therefore, we included a continuous measure as well as an instrument-specific expertise category. Given the mixed results from previous studies, the analysis whether these variables can predict the number of correct responses is exploratory.

Our study also contributes to the verification of knowledge in the social sciences and psychology, as addressed in the ongoing discussion on replicability of findings, the so-called “replication crisis” (Pashler and Wagenmakers, 2012; Open Science Collaboration, 2015). As addressed in more recent analyses of research practices in music psychology (Düvel and Altemeier, 2025; Eerola, 2025), there is a general lack of replication studies in this discipline: This type of publication reaches a share of only 3% (Eerola, 2025) and 3.8% (Düvel and Altemeier, 2025) of total publications in the domain of music psychology. Accordingly, our study also contributes to the demand for transparency by making all stages of research—such as methods, data, software, and reports—accessible to other researchers and the public (see Data Availability Statement).

## 2 Methods

### 2.1 Stimuli

We searched for suitable video recordings available from international violin competitions. As outlined in Section 1.3, the three performances of a competition required for a 3-AFC task should be equivalent in terms of the piece of music and the camera perspective and movement.

Many music competitions have a mandatory repertoire for the preliminary rounds, whereas contestants have more freedom in the selection of pieces in the final round. Given that this makes it unlikely to find suitable material, we decided to look at the semi-finals. In semi-finals, compulsory pieces generally remain or at least a limited pool from which to select. Regarding the piece of music, a competition was considered suitable provided at least two of the semi-finalists performed the same piece as the eventual winner in the same round.

Next, the video recordings of the identified competitions with suitable performances regarding the piece of music were screened for sections with equivalent camera perspective and movement. Unlike Tsay (2013), we deliberately decided to use clips containing a musically meaningful unit (e.g., a phrase or motif) instead of clips fixed to a 6-s length. Our intention was to maintain consistent musical content in the clips from the same competition, as variations in tempo and expressive timing are not accounted for when using a fixed absolute length.

This process resulted in four international music competitions with suitable video recordings from their semi-finals. Table 1 details the material from the identified competitions used as stimuli.

**Table 1:** Stimulus materials.

Competition	Excerpt	Camera	Contestants	Length
Joseph Joachim Violin Competition 2021, Hannover, Germany	Wolfgang Amadeus Mozart: Violin Concerto No. 5 A Major, 1st Movement *Allegro aperto* (K219), Bars 176–186	Medium/shoulder close up	**Maria Ioudenitch**, Elli Choi, Minami Yoshida	21–22 s
Menuhin Competition 2021, Richmond, VA, USA	Mark O’Connor: Menuhin Caprice, Bars 1–20	Medium long shot	**María Dueñas**, Karisa Chiu, Serin Park	21–24 s
Odesa International Violin Competition 2021, Odesa, Ukraine	Oleksandr Shymko: Molto ritmico, Bars 1–12	Long shot	**Dmytro Udovychenko**, Bohdan Shalyh, Anna Tanaka	24–25 s
Queen Elisabeth Competition 2019, Brussels, Belgium	Wolfgang Amadeus Mozart: Violin Concerto No. 5 A Major, 1st Movement *Allegro aperto* (K219), Bars 40–41	Medium/shoulder close up	**Stella Chen**, Daniel Kogan, Christine Lim	16 s

Contestants printed in bold are the respective winners.

The videos were retrieved from publicly available sources (e.g., YouTube or the website of a competition). Clips were created with the video editing software DaVinci Resolve^2^. The names of the performers were displayed in the videos of one of the competitions (Menuhin Competition 2021). To avoid them being used as possible cues, names were blurred. All audio tracks were normalized to −20 LUFS and a fade in and fade out was added with the Audacity^3^ software. The clips were exported in three versions: first, as audio files for the audio-only condition; second, as video files with an audio track for the audiovisual condition; and third, as video files without any audio track for the video-only condition.

### 2.2 Measures

As in Tsay’s (2013) original study, the dependent variable was the participants’ performance in the 3-AFC task “who wins the competition” in the three conditions as measured by the number of correct responses.

To assess participants’ musical background we used the Goldsmiths Musical Sophistication Index (Gold-MSI; Müllensiefen et al., 2014) in the validated German translation by Schaal et al. (2014). The Gold-MSI comprises 38 self-report items structured in a general factor of musical sophistication and the five subscales Active Engagement, Musical Training, Emotions, Singing Abilities, and Perceptual Abilities. To reduce the risk of participants dropping out due to the length of the questionnaire, we only used the 18 items of the General Factor and the nine items of the Perceptual Abilities subscale resulting in 25 items as two items are shared.

Alongside the 3-AFC task of identifying the winner of a triad, participants also rated each performance on the Performance Evaluation Scale (PES; Kopiez et al., 2017). The PES consists of four items determined by a Rasch model. The items (e.g., “The performance was authentic. [Die Performance war authentisch.]”) are answered on a 4-point Likert-scale (1 = *not at all* [*gar nicht*], 2 = some*what* [*etwas*], 3 = *quite a bit* [*ziemlich*], 4 = *very much* [*sehr*]). The PES score is the number of responses that are either 3 or 4, and thus ranges from 0 to 4.

### 2.3 Procedure

The study was conducted online on the SoSci Survey platform (Leiner, 2023). Data were collected from October 2023 to February 2024. The first page of the questionnaire provided information about the study, outlining the task and the three conditions. It also included the technical requirements, stated that participants would also have to answer questions about themselves, and that participation would take about 20 min. After giving their informed consent with a check box, participants provided their age, gender, and educational level. They also indicated their expertise in playing the violin or the viola (“Do you play the violin or viola, or have you ever played the violin or viola?,” 1 = *No*, 2 = *Yes, as an amateur. I only play my instrument as a hobby.*, 3 = *Yes, semi-professionally. I am sometimes paid for playing my instrument.*, 4 = *Yes, professionally. I earn my living from it.*). Participants then answered the 25 items of the Gold-MSI. In between these items, an instructed response item (“Please select ‘Strongly agree’”) was used as this provides a criterion to filter out inattentive participants with potentially meaningless data (Leiner, 2019). Twenty-two Gold-MSI items that share the same response scale and the instructed response item were administered in a random order, distributed across four questionnaire pages.

Before the main part of the study, participants were presented with one sample clip per condition (i.e., one audio clip, one video clip with sound, one video clip without sound). These clips derived from a music competition that was not considered for the set of actual stimuli but matched their recording quality. In addition to demonstrating the three conditions, the clips served to adjust a comfortable volume level. After the sample clips, participants ranked the three presentation conditions in order of priority for evaluating music performances. They were then informed that they would be presented with six triads of performances from violin competitions in the three conditions, and that they would have to rate the performances and identify the eventual winner in each triad.

**Figure 1:**
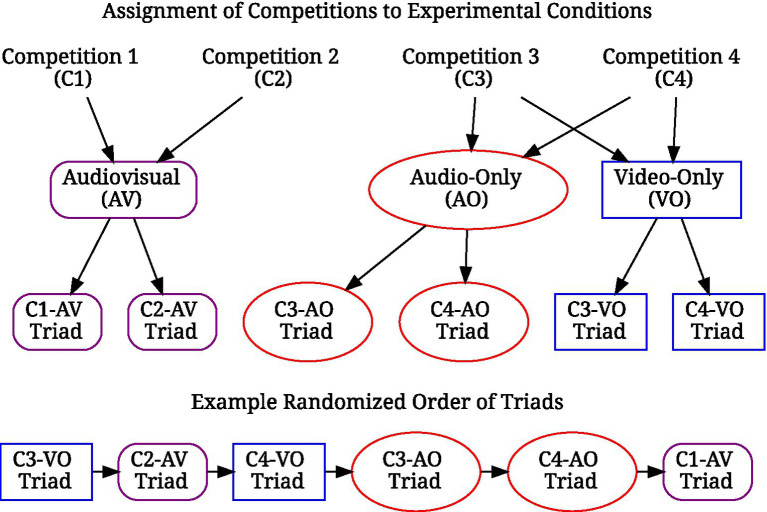
Stimulus randomization process. The video and audio tracks of each competition were presented only once. For the two competitions assigned to the audiovisual condition, both tracks were presented simultaneously. For the remaining two competitions, video and audio were presented separately in the video-only and audio-only conditions, respectively.

During the main part of the study, participants were presented with two of the triads in the audiovisual condition and the remaining two triads in both the audio-only and video-only conditions. This way, participants would (though not necessarily simultaneously) see and hear each triad only once, which would prevent responses from being carried over between conditions (i.e., when a competition triad was presented in the audiovisual condition, it was not presented in the video-only and audio-only conditions, and vice versa). This also resulted in an incomplete 3 (conditions) × 2 (triads) within-subjects design. The four competitions were assigned to the three conditions randomly for each participant (e.g., the Joseph Joachim Violin Competition and Menuhin Competition triads are assigned to the audiovisual condition, and the Odesa International Violin Competition and Queen Elisabeth Competition triads are assigned to both the audio-only and video-only condition). The presentation order of the resulting six triads (two per condition), as well as the order of performances within a triad was determined randomly for each participant. Figure 1 illustrates the randomization of the stimuli. The questionnaire page for a competition in one of the experimental conditions contained the media player and the PES items for each performance below each other. At the bottom of the page, the question of “who would win the competition” was placed.

Once the main part of the study was complete, the participants filled in another instructed response item and indicated how easy they had found it to make their decisions in each of the three conditions. They were then thanked, and the study was terminated.

### 2.4 Data analysis

#### 2.4.1 Performance in the 3-AFC task of identifying the winner

Response data for each condition were modeled within the framework of Signal Detection Theory (SDT) and Sensory Discrimination Measurement. In this framework, the ability to discriminate entities belonging to two distinct categories is measured. In the classical SDT experiment, participants have to decide whether a signal is present or absent in background noise (Hautus et al., 2021). Besides this Yes–No or A–Not A design, SDT provides an entire family of designs and is used in different fields.

For this study, we define competition winners as the signal category. The no-signal category comprises performers who were not selected as winners by the jury. Therefore, identifying the winner represents a discrimination task. As participants were presented with two triads per condition, the discrimination task is replicated in terms of SDT (Bi, 2015; Hautus et al., 2021). Data from replicated discrimination tasks is modeled with a corrected beta-binomial model (CBB; Bi, 2015, chap. 10). This model assumes that the probability for a correct response, *p*_c_, is determined by the probability of guessing correctly, *p*_0_, which is 1/3 in a 3-AFC task, and the discrimination ability quantified as d prime, *d*′. Furthermore, the model assumes participants to have different discrimination abilities. If participants have the same discrimination ability, the data follows a binomial distribution with probability parameter greater than *p*_0_ instead of a corrected beta-binomial distribution. If participants have no discrimination ability, that is, *d*′ = 0, the data follows the guessing model, namely, a binomial model with probability parameter equal to *p*_0_.

Bi (2015, chap. 10.4) provides a *Z* statistic to test whether the mean of the true discrimination ability of the participants is greater than 0. In addition to the mean of the true discrimination ability, the statistical power of this test is a function of the dispersion of discrimination abilities among participants, the number of participants, the number of replications, and the α level (Bi, 2015, chap. 10.5). We estimated the dispersion of discrimination abilities from the data provided by Mehr et al. (2018) by fitting a CBB model for the video-only condition from their first experiment (γ = 0.326). Based on this estimate, the frequencies of correct responses reported by Tsay (2013, Experiments 2–5) and Mehr et al. (2018, Experiment 1) as estimates for the mean discrimination ability, α = 0.05, and a power of 1 − β = 0.80, we calculated scenarios for the required sample size. Table 2 shows the required number of participants for the different scenarios. As we wanted to compare the three conditions with an equal number of participants, using four replications—that is, presenting all four competition triads in one condition—would have resulted in a between-subjects design, meaning the total sample size would have been thrice the sample size for one condition. Therefore, the incomplete within-subjects design with two replications per condition was a pragmatic choice to reduce the possibly required total sample size.

**Table 2:** Sample size scenarios based on the relative frequencies of correct responses in the video-only condition from previous studies and different numbers of replications.

Study	Correct responses	Replications	Required Participants	
			Per Condition	In Total
Tsay (2013) Experiment 2	0.5250	2	21	21
Tsay (2013) Experiment 3	0.4640	2	44	44
Tsay (2013) Experiment 4	0.4660	2	42	42
Tsay (2013) Experiment 5	0.4700	2	40	40
Mehr et al. (2018) Experiment 1	0.3867	2	251	251
Tsay (2013) Experiment 2	0.5250	4	11	33
Tsay (2013) Experiment 3	0.4640	4	23	69
Tsay (2013) Experiment 4	0.4660	4	23	69
Tsay (2013) Experiment 5	0.4700	4	21	63
Mehr et al. (2018) Experiment 1	0.3867	4	130	390

The SDT analyses were carried out by means of the sensR R package (Christensen et al., 2023) that identifies discrimination tests as generalized linear models (Brockhoff and Christensen, 2010), and based on Bi (2015).

#### 2.4.2 Task performance, musical sophistication, and expertise

To determine whether General Musical Sophistication, the Perceptual Abilities subscale, or the violin/viola expertise can predict the task performance, a beta-binomial regression was calculated per condition by means of the VGAM R package (Yee and Wild, 1996; Yee, 2015; Yee and Moler, 2025). Since the General Musical Sophistication factor and the Perceptual Abilities subscale are not independent and share items, they were not used as predictors directly. First, they were standardized to *z* scores. Second, Perceptual Abilities *z* scores, *z*_PA_, were linearly regressed on General Musical Sophistication *z* scores, *z*_GMS_. By definition, the resulting residuals are not correlated with the standardized General Musical Sophistication scores and thus represent the Perceptual Abilities corrected for General Musical Sophistication. Equation 1 shows the predictor structure for the beta-binomial regression.


$$\begin{array}{l} \text{Correct Responses}∼z_{\mathrm{GMS}}+\text{Residuals}\;(\mathrm{lm}(z_{\mathrm{PA}}∼z_{\mathrm{GMS}}))\\ \;+\text{Violin}/\text{Viola Expertise} \end{array}$$ (1)

### 2.5 Participants

Participants were recruited through university mailing lists and personal contacts in Germany. The link to the online study was clicked 496 times. Of these, the questionnaire was completed 135 times. Alongside the instructed response items, a minimum time of 11 min for the questionnaire was defined as a filter criterion. A total of *N* = 104 passed these criteria. With this sample size, the planned *Z* test has a power of 1 − β = 0.495 when estimating the discrimination ability according to Mehr et al. (2018; *p*_c_ = 0.387) and a power of 1 − β ≥ 0.983 when estimating the discrimination ability according to Tsay (2013; *p*_c_ ≥ 0.464).

Participants were aged 19–73 years (*M* = 32.04, *SD* = 13.11; 55 female, 47 male, and 2 with gender undisclosed). Regarding the participants’ violin or viola expertise, 6 reported being professional players, 6 semi-professional, 22 amateur, and 70 not having played the violin or viola. Musical sophistication was slightly above average compared to the sample by Schaal et al. (2014). Scores on the General Musical Sophistication factor (*M* = 87.43, *SD* = 21.47) fell into the 65th percentile, and on the Perceptual Abilities subscale (*M* = 51.02, *SD* = 8.76) into the 70th percentile.

### 2.6 Ethical approval statement

The study was performed in accordance with relevant institutional and national guidelines (Föderation Deutscher Psychologenvereinigungen, 2022; Präsidium der Hochschule für Musik, Theater und Medien Hannover, 2024) and with the principles expressed in the Declaration of Helsinki.

## 3 Results

### 3.1 Performance in the 3-AFC task of identifying the winner

**Figure 2:**
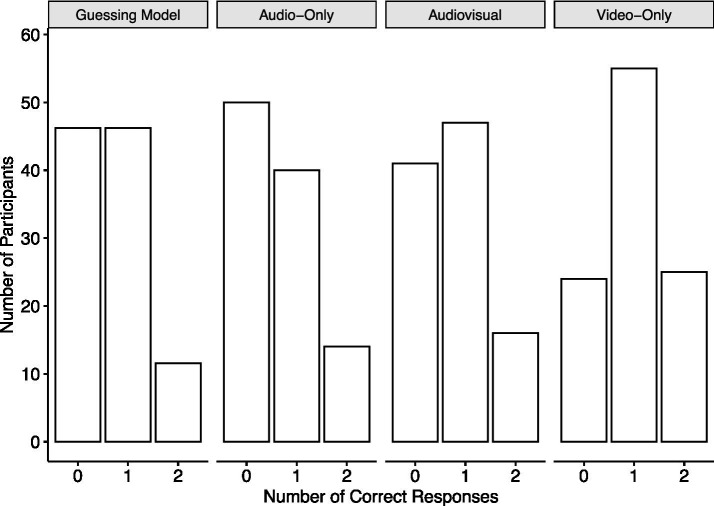
Number of participants achieving 0, 1, or 2 correct responses according to the expectation of the guessing model and in the three experimental conditions.

According to the guessing model for two triads, the probabilities of a participant having 0, 1, or 2 correct responses are (1 − *p*_0_)^2^ = 4/9, 2*p*_0_(1 − *p*_0_) = 4/9, and *p*_0_^2^ = 1/9, respectively. The first column of Figure 2 shows the number of participants who should achieve 0, 1, or 2 correct responses based on the expectation of the guessing model. Figure 2 also shows the number of participants who actually achieved 0, 1, or 2 correct responses in the three experimental conditions. While the bar diagrams for the audio-only and the audiovisual conditions resemble that of the guessing model, upon visual inspection, the video-only condition is different. Testing the proportion of correct responses of 0.505 against *p*_0_ = 1/3 as described by Bi (2015, chap. 10.4), results in a significant difference from chance, *Z* = 5.25, *p* < 0.001. A likelihood ratio test comparing the CBB model and the guessing model on the data of the video-only condition favors the CBB model, *G*^2^ = 25.9, df = 2, *p* < 0.001. The population level discrimination ability is *d*′ = 0.572. Neither the audio-only condition with a proportion of 0.351 correct responses, *Z* = 0.54, *p* = 0.295, *G*^2^ = 0.549, df = 2, *p* = 0.760, nor the audiovisual condition with a proportion of 0.38 correct responses, *Z* = 1.42, *p* = 0.078, *G*^2^ = 2.151, df = 2, *p* = 0.341, differ significantly from the guessing model.

A one-way repeated measures ANOVA with the three experimental conditions as independent variable and the number of correct responses as dependent variable revealed differences between the conditions, *F*(2, 206) = 7.47, *p* < 0.001, η_p_^2^ = 0.07. As indicated by Holm-corrected *post hoc* tests, the proportion of correct responses was higher in the video-only condition than in the conditions involving audio (see Table 3).

**Table 3:** Holm-corrected pairwise comparisons of the experimental conditions.

Contrast	Estimate	*SE*	df	*t*	*p*
Audio-Only vs. Audiovisual	−0.053	0.049	103	−1.074	0.285
Audio-Only vs. Video-Only	−0.178	0.046	103	−3.885	< 0.001
Audiovisual vs. Video-Only	−0.125	0.047	103	−2.676	0.017

### 3.2 Task performance, musical sophistication, and expertise

In the video-only and audiovisual conditions, none of the predictors proved significant. In the audio-only condition, the Perceptual Abilities residuals, *b* = 1.039, *SE* = 0.4109, *z* = 2.529, *p* = 0.011, as well as being a professional violinist or violist compared to not playing or having played one of the instruments, *b* = 1.783, *SE* = 0.6822, *z* = 2.613 *p* = 0.009, turned out as significant positive predictors.

### 3.3 Relationship between PES ratings and 3-AFC responses

An exploratory analysis of the relationship between PES ratings and the responses to the 3-AFC tasks was conducted. Participants rated each performer from a triad on the PES and then selected one of them as the eventual winner. Table 4 displays the frequencies for the cases in which participants gave the actual winner their highest PES rating and in which their selected performer in the 3-AFC task received their highest PES rating. As shown, participants’ selected winner almost always received their highest PES score. The actual winners received the highest PES scores about 2/3 of the time. However, the fact that participants were allowed to give more than one performer in a triad their highest PES rating should be noted. Therefore, two or even all performers within a triad may receive the same score from a participant.

**Table 4:** Frequencies of the cases in which participants gave their highest PES score to their 3-AFC answer and the actual winner.

Condition	*n*	Frequency winner has PES maximum		Frequency 3-AFC answer has PES maximum	
		Absolute	Relative	Absolute	Relative
Audio-Only	208	136	0.65	201	0.97
Video-Only	208	148	0.71	194	0.93
Audiovisual	208	138	0.66	193	0.93

## 4 Discussion

This study aimed to conceptually replicate the SOS effect, first observed by Tsay (2013), however, based on stimulus material controlled for musical content and camera perspective and movement. The main finding of the original study was successfully replicated as participants were able to identify the winner of a competition in a triad of three silent videos above chance level but performed at chance level with triads of audio clips and triads of audiovisual videos.

Musical sophistication or violin/viola expertise did not predict the performance in the video-only and the audiovisual conditions which is consistent with the findings by Tsay (2013) and Mehr et al. (2018). Unlike these previous studies, in our study perceptual abilities and being a professional violinist or violist were significant predictors for the number of correct responses in the audio-only condition. While it is plausible that domain-specific experts can discriminate two very similar categories based on what they hear (e.g., electric guitar amplifiers and their simulations; Düvel et al., 2020), which should be true for the jury and the reason why the winners became the jury’s winner, there were only 6 professional violinists and violists in our sample compared to 70 participants with no expertise in the violin and the viola.

Considering the exploratory analysis of the relationship between the PES ratings and the 3-AFC responses, where the performance selected to be the eventual winner almost always received the highest rating within a triad, it could be surmised that participants assumed that the original jury had come to the same or very similar ratings. In other words, participants could have believed that judges valued the same things or used the same criteria as them. Although not statistically tested, there is a trend that the actual winners received the highest PES rating in the video-only condition (71.2%) more often than in the audio-only (65.4%) and audiovisual conditions (66.3%, see Table 4). This is similar to the results from Tsay’s (2013) Experiment 7. Whereas Tsay’s participants had to choose one performer from the triad who demonstrated each of the six provided adjectives most strongly, participants in the present study were allowed to give the same PES rating to two or all three performers within a triad.

### 4.1 Limitations and future directions

Although the stimulus material was controlled for musical content, and camera perspective and movement, some unavoidable variability in the triads remains. This partly reflects the fact that the camera is equivalent but not identical within a triad. For example, in the Menuhin Competition triad, although the camera perspective and movement are very similar, at least one performance is situated in a different room. Moreover, when performers are accompanied by a pianist or an ensemble, these musicians introduce additional variability. The Odesa Competition triad shows only the soloists whereas the remaining three competition triads display additional musicians. This remaining variability in the stimulus material was accepted because material was difficult to find that matched our criteria both invariance in musical content and camera.

Limited stimulus material also resulted in the presentation of only two triads per condition. Alternatively, using a between-subjects design would have doubled the number of triads per condition. However, Samma et al. (2025) stated that their use of a between-subjects design rather than a within-subjects design was a limitation of their study. Using a between-subjects design would indeed have reduced the number of participants per condition to one third resulting in about 140 data points instead of the achieved 208. Although the number of triads—replications in terms of SDT—can partially compensate for a lack of participants this is limited even in the theoretical case of having an infinite number of replications (Bi, 2015, chap. 10.5).

Accordingly, future studies on the SOS effect should thoroughly control the stimulus material. While we were able to draw on violin competition recordings up to the year 2023, new suitable material may have been created in the meantime. Beyond using more stimulus material for the same instruments to increase the number of triads, a stimulus set for different instruments could be used to further explore the generalizability of the SOS effect as attempted by Chiba et al. (2023) and Samma et al. (2025). There may also be opportunities to collaborate with competitions, enabling direct involvement in the recording process and experimental manipulation of subsequent stimuli.

Following the repeated observation of the SOS effect, future studies should seek to explain the phenomenon. For example, by analyzing the stimulus clips and drawing on wide-ranging literature on the visual aspects of music performances, it should be possible to identify the underlying visual cues or features that enable the above-chance performance in the video-only condition. Davidson (1993) concluded that “vision can be more informative than sound in the perceiver’s understanding of the performer’s expressive intentions” (p. 112). These expressive intentions might meet potential expectations of the listeners in the auditory and especially in the visual domain (Juchniewicz, 2008). Performers’ movements represent a visual interpretation of the music that may also highlight structural features in addition to the expressive information (Behne and Wöllner, 2011). Regarding quantity of motion, Davidson (1993) and Juchniewicz (2008) found ratings increasing as a function of quantity of motion. In the study by Moura et al. (2024) utilizing two excerpts in five different degrees of motion, one excerpt followed the same pattern. For the second excerpt, specific gestures with varying degrees of motion seemed to be more important for the evaluation than the extent of movement alone. Based on their results on musical duo performances, Küssner et al. (2020) conclude that “certain visuokinematic cues attract observers’ visual attention, independent of the musical cues” (p. 207) and that “visuo-kinematic cues of performers moving more expressively likewise captured more of observers’ attention, without observers necessarily being aware of or able to control this behavior” (pp. 207–208). Like Juchniewicz (2008), they also emphasize the role of expectations which play an important part for the criterion of “appropriateness” (Platz and Kopiez, 2022).

There is also evidence that performers are—at least partially—aware of extra and non-musical factors in evaluating performances. Interviewing nine expert classical pianists, Urbaniak and Mitchell (2024) found that these pianists conceptualize their performances theatrically as evidenced by statements like “It’s an act—it’s a performance—we’re actors, and we’re on stage and it’s a drama” (P2, p. 236). This ties in with Goffman’s (2022) use of theater as a model. Turning to the domain of rock music, there are similar conceptions of the live performance among electric guitarists potentially including specific—sometimes technically dispensable—playing techniques (Lehmann and Kopiez, 2013; Platz and Kopiez, 2022). In their study of rock guitar solos, Lehmann and Kopiez (2013) found an effect of these live techniques and of visual engagement on impressiveness which in turn might result in a better evaluation of a performance.

Taken together, motion capture analysis of video material complemented by expert opinions from the fields of music as well as theater could be a promising approach for the development of such a model of cue extraction.

### 4.2 Conclusion

Drawing on previous studies and our replication, we conclude that the SOS effect should be considered as a valid finding and should be considered in future models of performance evaluation. Following Platz and Kopiez (2022), we recommend that musicians not only prepare for a shining rendition of a composition or improvisation in the auditory domain, but also develop an awareness for additional aspects of the performance—particularly, the visual component—or put differently to conceptualize “performance as a multimodal spectacle … to maximize … performance success” (Urbaniak and Mitchell, 2024, p. 240). Using video recordings of one’s own presentations or consulting an acting coach could be promising approaches. For example, as Platz and Kopiez (2013) and Waddell and Williamon (2017) could show, performance evaluation already starts with a performer’s stage entrance behavior.

## Data Availability

The stimuli, data set, and analysis code are available on osf: https://osf.io/ynved.

## Footnotes

1 Tsay (2013, 2014) and the authors of the previous replication studies do not report effect sizes for all of their analyses. Therefore, only the effect sizes provided by the original authors are presented here.

2 DaVinci Resolve Version 18.6, https://www.blackmagicdesign.com/products/davinciresolve

3 Audacity Version 3.3.3, https://www.audacityteam.org/
